# Sequence analysis of *MYOC* and *CYP1B1* in a Chinese pedigree of primary open-angle glaucoma

**Published:** 2011-05-31

**Authors:** Jun Chen, Su-ping Cai, Wenhan Yu, Naihong Yan, Li Tang, Xiaoming Chen, Xuyang Liu

**Affiliations:** Ophthalmic Laboratories and Department of Ophthalmology, West China Hospital, Sichuan University, P.R. China

## Abstract

**Purpose:**

To analyze two candidate genes, trabecular meshwork inducible glucocorticoid response (*MYOC*/*TIGR*) and human dioxin-inducible cytochrome P450 (*CYP1B1*), in a Chinese pedigree of primary open-angle glaucoma.

**Methods:**

In a three-generation family containing 14 members, four of them were patients with primary open-angle glaucoma, one was a glaucoma suspect, and the rest were asymptomatic. All members of the family underwent complete ophthalmologic examinations. Exons of *MYOC* and *CYP1B1* were amplified by polymerase chain reaction, sequenced, and compared with a reference database.

**Results:**

Elevated intraocular pressure and impaired visual field were found in all patients. One *MYOC* heterozygous mutation G367R, in exon 3 was identified in four patients and the suspect, but not in the rest of the family members. Meanwhile, four single nucleotide polymorphisms in *MYOC* and *CYP1B1* genes were found.

**Conclusions:**

Although the G367R mutation of *MYOC,* which causes primary open-angle glaucoma in the form of autosomal dominant inheritance, has been reported in some other ethnicities, it was found in Chinese pedigree for the first time.

## Introduction

Glaucoma is one of the leading causes of blindness in the world and is characterized by optic disc cupping and visual field defects [1,2]. Primary open-angle glaucoma (POAG) is the most common form of glaucoma [3]. There are two forms of POAG: juvenile onset and adult onset. Usually, juvenile open angle glaucoma (JOAG) may manifest clinically between the ages of 3 and 30 [4,5], while adult POAG manifests clinically after the age of 40 [6,7]. Although the exact mechanisms of POAG remain unclear, the accumulating evidences suggest that the genetic basis plays an important role in its pathogenesis. Four genes, trabecular meshwork inducible glucocorticoid response (*MYOC*/*TIGR*), human dioxin-inducible cytochrome P450 (*CYP1B1*), optineurin (*OPTN*), and WD repeat domain 36 (*WDR36*), have been identified as glaucoma-causing genes [8], with *MYOC* being the first identified POAG gene [9]. To date, more than 70 mutations have been detected in *MYOC* worldwide [10]. About 90% of the mutations were located in exon 3 where the olfactomedin-like domain is located [11]. Recently, *CYP1B1* has been shown to be related to POAG, especially JOAG [12-14]. Both *MYOC* and *CYP1B1* consist of three exons, but in *CYP1B1*, only exon 2 and 3 encode the protein.

In this study, alterations in *MYOC* (three exons) and *CYP1B1* (exon 2 and 3) were analyzed, and a known mutation (c.1099 G>A, G367R) in exon 3 of *MYOC*, which was segregated with the disorder within the family and appeared to be the disease-causing gene, was found. It is for the first time, to the best of our knowledge, that G367R mutation was found in Chinese.

## Methods

### Clinical examination

**Figure 1:**
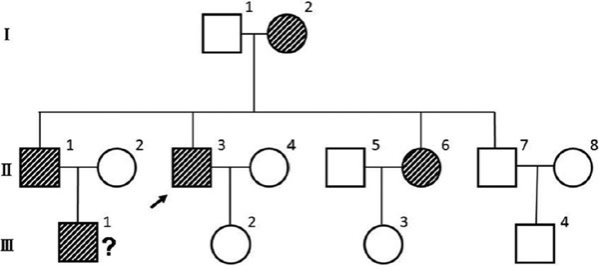
Pedigree for the Chinese POAG family. The proband was II-3.

This three-generation pedigree with POAG (Figure 1) was recruited from the out-patient department of Ophthalmology at West China Hospital (Sichuan University, Chengdu, P. R. China). All members of the family underwent the complete ophthalmologic examinations including slit-lamp biomicroscopy, gonioscopic examination, fundoscopic examination, IOP measurement (Canon TX-F Non-contact tonometer; Canon Inc., Tokyo, Japan), and visual field test (Octopus 900; HAAG-STREIT International, Berne, Swiss). Diagnostic criteria for POAG included open anterior chamber angle, elevated IOP (≥22 mmHg), glaucomatous visual field defects and characteristic optic disc damage.

The study was approved by the medical ethics committee of the West China Hospital of Sichuan University. This study follows the principles of the Declaration of Helsinki. All subjects were clinically evaluated by glaucoma specialists.

### Detection of *MYOC* and *CYP1B1* mutations

Genomic DNA was extracted from 0.2 ml peripheral blood sample with Qiamp Blood Kit (Qiagen, Hilden, Germany) according to the manufacturer’s instruction.

Intronic primers flanking the exons were designed (Table 1) based on gene sequences of *MYOC* (GenBank AF001620) and *CYP1B1* (GenBank U56438) and synthesized by Invitrogen (Carlsbad, CA). PCR amplification was performed in a MyCycler thermocycler (Bio-Rad, Hercules, CA). The 30 μl PCR reaction mixtures included 30 ng DNA, 1× PCR buffer, 2.5 mM MgCl_2_, 0.3 mM of each of dNTPs, 1.5 U Pfu DNA polymerase, and 1.0 μM each of the forward and reverse primers. All reagents used in this procedure were purchased from TaKaRa (Dalian, China). The reactions were incubated at 95 °C for 4 min followed by 35 cycles at 95 °C for 30 s, 58 °C for 30s, and 72 °C for appropriate time (the second exon of *MYOC* for 30s and the rest for 90s), and then a final extension at 72 °C for 5 to 10 min.

**Table 1:** Primers used in PCR for amplification of *MYOC* and *CYP1B1*.

**Exons**	**Primer sequence (forward/reverse)**	**Product size (bp)**
*MYOC* 1	PF 5′-CCAAACAGACTTCTGGAAGG-3′	904
*MYOC* 1	PR 5′-TAGCAGGTCACTACGAGCC-3′	
*MYOC* 2	PF 5′-TGTCATCCTCAACATAGTCA-3′	351
*MYOC* 2	PR 5′-TTCTGTTCCTCTTCTCCTC-3′	
*MYOC* 3	PF 5′-CCAGGGCTGTCACATCTACT-3′	933
*MYOC* 3	PR 5′-CATCTCCTTCTGCCATTGC-3′	
*CYP1B1* 2	PF 5′-CATTTCTCCAGAGAGTCAGC-3′	1260
*CYP1B1* 2	PR 5′-GCTTGCAAACTCAGCATATTC-3′	
*CYP1B1* 3	PF 5′-ACCCAATGGAAAAGTCAGCC-3′	927
*CYP1B1* 3	PR 5′-GCTTGCCTCTTGCTTCTTATT-3′	

PCR products were directly sequenced by an ABI 377XL automated DNA sequencer (Applied Biosystems, Foster City, CA). Sequence data were compared pair-wise with the published *MYOC* and *CYP1B1* sequences.

## Results

### The proband and other patients (Table 2)

**Table 2:** Patient data from this POAG pedigree

**Patient**	**Age at study (years)**	**Diagnosis age (years)**	**Operation age (years)**	**Maximal IOP (mmHg)**	**C/D ratio**	**Visual field damage**
I-2	68	38	41	>50 (ou)	0.95/1.0	Severe
II-1	48	30	34	50~60 (ou)	0.9/0.95	Moderate
II-3	46	46	—	28.9 (od), 28.1 (os)	0.5/0.6	Moderate
II-6	42	20	24	50 (od), 65 (os)	0.9/0.95	Severe
III-1	25	25	—	24.4 (od), 23.6 (os)	0.2	Normal

**Figure 2:**
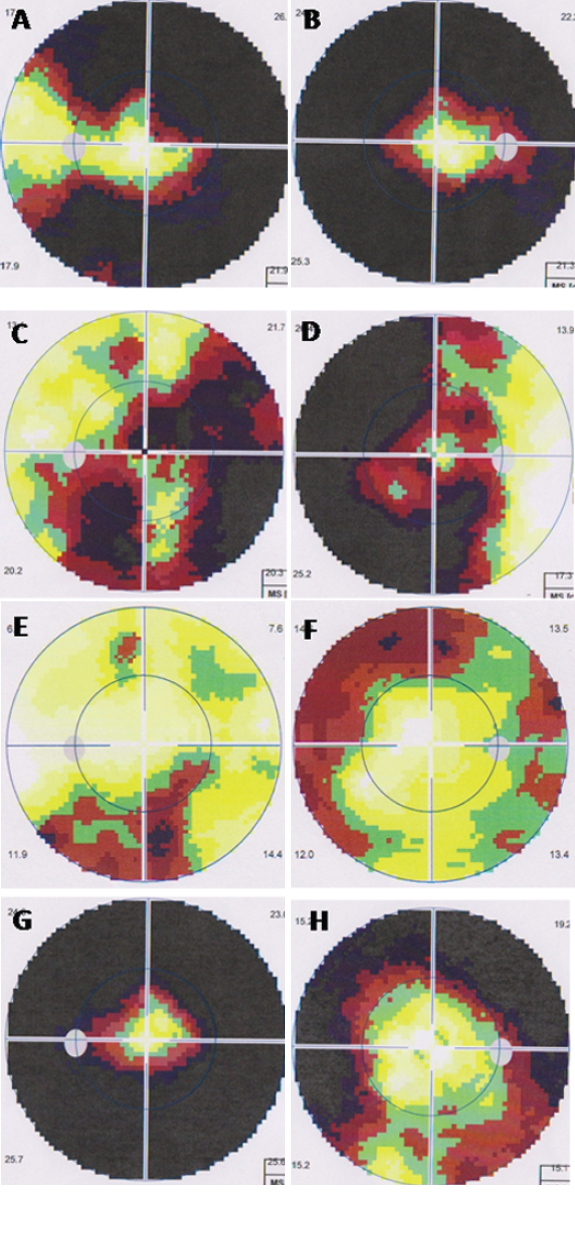
Visual fields of patients. Visual field of I-2 (**A**, **B**), II-1 (**C**, **D**), II-3 (**E**, **F**), and II-6 (**G**, **H**).

The proband (II-3) was diagnosed with POAG (both eyes) at the age of 46, with elevated IOPs (26.7 mmHg in the right eye and 23.1 mmHg in the left eye), open anterior chamber angle, enlarged cup-disc ratio of 0.5/0.6(OD/OS) and characteristic glaucomatous visual field defects (Figure 2). Other ocular abnormalities or systemic disorders were not found.

The proband’s mother (I-2) was diagnosed with POAG at the age of 38, and trabeculectomy was performed for both eyes twenty-one years ago. During this examination, a cup/disc ratio of 0.95/1.0 (OD/OS), IOPs at 16.7/19.1 mmHg (OD/OS), and late-stage glaucomatous visual field loss were noticed (Figure 2). II-1 was a JOAG patient, and bilateral trabeculectomies were performed at the age of 34, when his IOPs were as high as 50~60 mmHg (OU). Patient II- 6 's onset of the glaucoma was at the age of 20, much earlier than other family members. Her maximal IOPs measured 50 mmHg in the right eye and 65 mmHg in the left eye. She underwent trabeculectomy in both eyes. The cup-disc ratio of both II-1and II-6 was 0.9/0.95 (OD/OS). Patient III- 1, 25 years old, was a glaucoma suspect, because he had a strong family history of glaucoma and his IOP measured 27.8/21.5 mmHg (OD/OS), despite no glaucomatous visual field defects and characteristic optic disc damage.

### Asymptomatic family members

The proband’s father (I-1) didn’t have ocular diseases except senile cataract. No ocular abnormalities were found in the rest of the family numbers. Their visual acuity or corrected visual acuity was measured ≥20/20.

### Sequencing results

#### MYOC

**Figure 3:**
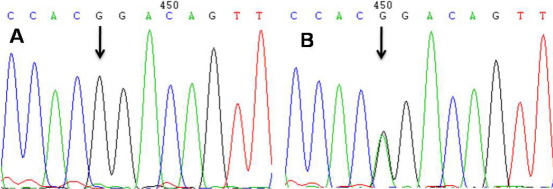
*MYOC* mutation in the POAG family. **A**: Normal individuals with homozygous G (arrow). **B**: The double peak of guanine (black line) and adenine (green line; **B**, arrow) represents a heterozygous mutation at the codon of 367th amino acid residue (Gly367Arg).

Sequence analysis of *MYOC* revealed a heterozygous mutation, c.1099G>A (G367R), in exon 3 in all patients and the suspected one but not in any of the asymptomatic members of the family. The G367R *MYOC* mutation was cosegregated with the disorder within the family (Figure 3). One single nucleotide polymorphism (SNP, g.23096344C>T) in exon 2 of *MYOC* was identified.

#### CYP1B1

No mutation was found. Three *CYP1B1* SNPs (g.17120037A>G, g.17120090C>G, and g.17120026T>C) were identified in exon 3.

## Discussion

*MYOC* was the first identified POAG gene [9]. Previous studies showed that *MYOC* mutations exist in nearly 3% of adult onset POAG patients and a greater proportion of JOAG patients [15,16]. In this study, a G to A transition at the first base of codon 367 (in exon 3 of *MYOC*), which resulted in a glycine to arginine amino acid substitution, was identified, suggesting that *MYOC* is the glaucoma-causing gene in this family. This mutation has been previously reported in several other ethnic groups: Japanese, Indian, Irish, Swiss, French-Canadian, Scottish, and German [17-25], however, to the best of our knowledge, this mutation was found in Chinese for the first time.

Based on the literature reviewed, the phenotype of POAG associated with the G367R mutation was summarized in Table 3: 1) there were no obvious differences between gender; 2) all carriers had open anterior chamber angle; 3) all 6 pedigrees were in autosomal dominant fashion; 4) the patients had relatively high IOP and relatively early onset age; and 6) medical control of IOP were not satisfied in most of the patients, and surgeries were usually needed. The Chinese pedigree with G367R mutation reported in this paper was in general coincidence with the characteristics mentioned.

**Table 3:** Glaucoma phenotype of patient with myocilin G376R mutation.

**Ethnic origin**	**Type of glaucoma**	**Carrier number**	**Maximum IOP (mmHg)**	**Age at diagnosis (year)**	**Hereditary pattern**	**Publication year**
Japanese	POAG	1	ND	45	ND	1997
Irish	POAG	5	ND	ND	AD	1998
German	JOAG	2	36	14 & 21	AD	1998
Japanese	POAG & suspect	8	50	36.7 (average age)	AD	2000
French-Canadian	Both	7	>50 (4/7)	34 (median age)	AD & Sporadic	2002
UK (Scottish)	JOAG	2	43 & 52	34 & 21	AD	2002
Indian JOAG	1	50.6	32	Sporadic	2003	
French	ND	1	ND	ND	Sporadic	2003
Swiss	Both & suspect	13	50	28–51	AD	2008

Abbreviations are as follows: AD, Autosomal Dominant; ND, Not Described.

The phenotypic variations observed here and in previous studies suggest that, in addition to G367R substitution, some as yet unidentified factors (such as the genetic and/or environmental) are responsible for the disease phenotype. Furthermore, POAG is well known to be genetically heterogeneous and several loci have been identified except *MYOC*. The variations in these loci may contribute to phenotypic variations. Recently, another gene *CYP1B1*, indeed, has been suggested to modify the glaucoma phenotype [8]. It may act as a modifier of *MYOC* expression or the two genes may interact via a common pathway [26,27]. We continued to screen *CYP1B1* gene, but no mutations, except three SNPs, were detected.

It is known that patients with missense mutations such as G367R are likely to present the normal and mutant mRNA in equal amounts, and then the mRNA is translated into an equal ratio of mutant and normal proteins. Myocilin was thought to be a secreted protein [28], but mutant Myocilin formed insoluble aggregates that could not be secreted out from human trabecular meshwork (HTM) cells and accumulated intracellularly, then damaged the function of HTM cells and resulted in an increase of aqueous humor outflow resistance [29-31]. So far, the secretion of G367R mutation myocilin protein has been studied in vitro and in vivo, which revealed the same mechanism above [32].

In conclusion, the G367R mutation of *MYOC* in this pedigree appears to be the cause of the disease in this family. This is the first time that this mutation was found in Chinese.
